# Arbuscular Mycorrhizal Fungi and Plant Growth-Promoting Pseudomonads Increases Anthocyanin Concentration in Strawberry Fruits (*Fragaria x ananassa* var. Selva) in Conditions of Reduced Fertilization

**DOI:** 10.3390/ijms140816207

**Published:** 2013-08-06

**Authors:** Guido Lingua, Elisa Bona, Paola Manassero, Francesco Marsano, Valeria Todeschini, Simone Cantamessa, Andrea Copetta, Giovanni D’Agostino, Elisa Gamalero, Graziella Berta

**Affiliations:** 1 Department of Sciences and Innovative Technology, University of Piemonte Orientale, Viale T. Michel 11, Alessandria 15121, Italy; E-Mails: guido.lingua@unipmn.it (G.L.); paola.manassero@unipmn.it (P.M.); francesco.marsano@unipmn.it (F.M.); valeria.todeschini@unipmn.it (V.T.); simone.cantamessa@unipmn.it (S.C.); andrea.copetta@unipmn.it (A.C.); elisa.gamalero@unipmn.it (E.G.); graziella.berta@unipmn.it (G.B.); 2 Mybasol srl, Via Gentilini, Alessandria 15121, Italy; E-Mail: mybasol@libero.it

**Keywords:** anthocyanin, high performance liquid chromatography (HPLC), arbuscular mycorrhizae, plant growth-promoting bacteria, low fertilization

## Abstract

Anthocyanins are a group of common phenolic compounds in plants. They are mainly detected in flowers and fruits, are believed to play different important roles such as in the attraction of animals and seed dispersal, and also in the increase of the antioxidant response in tissues directly or indirectly affected by biotic or abiotic stress factors. As a major group of secondary metabolites in plants commonly consumed as food, they are of importance in both the food industry and human nutrition. It is known that arbuscular mycorrhizal (AM) fungi can influence the plant secondary metabolic pathways such as the synthesis of essential oils in aromatic plants, of secondary metabolites in roots, and increase flavonoid concentration. Plant Growth-Promoting Bacteria (PGPB) are able to increase plant growth, improving plant nutrition and supporting plant development under natural or stressed conditions. Various studies confirmed that a number of bacterial species living on and inside the root system are beneficial for plant growth, yield and crop quality. In this work it is shown that inoculation with AM fungi and/or with selected and tested *Pseudomonas* strains, under conditions of reduced fertilization, increases anthocyanin concentration in the fruits of strawberry.

## 1. Introduction

Anthocyanins are a group of widespread natural phenolic compounds in plants. They are mainly detected in flowers and fruits (especially in berries) and are responsible for bright colours such as orange, red and blue [1]. In strawberries, anthocyanin concentration correlates well with the darkness of the fruits [2]. Strawberry has a simple anthocyanin profile with only a few major pigments. The total anthocyanin concentration in ripe fruits of strawberry was observed to vary between 200 and 600 mg/kg [2], but lower concentrations were also reported [3,4].

Anthocyanins are glycosides and acylglycosides (even if strawberry anthocyanins are reported to be non-acylated [2]) of anthocyanidins. Anthocyanidins vary with different hydroxyl or methoxyl substitutions in their basic structure, which is a flavylium cation [1]. Anthocyanins are believed to play different important roles such as in the attraction of animals and seed dispersal, and also in the increase of the antioxidant response in tissues directly or indirectly affected by biotic or abiotic stress factors [5]. As a major group of secondary metabolites in plants commonly consumed as food, they are of importance in both the food industry and human nutrition. Recently, increased attention has been given to their possible health benefits in preventing chronic and degenerative diseases, including heart disease and cancer [6]. These effects were partly attributed to their antioxidant capability [7,8].

It is known that beneficial soil microorganisms, like bacteria and arbuscular mycorrhizal (AM) fungi can influence the plant secondary metabolic pathways [9]. AM fungi, which establish mutualistic symbioses with the root systems of about 80% of land plant species, including the most important agricultural crops [10], are known to improve the plant mineral nutrition [10] and to modulate (often in a species-specific manner) the synthesis of essential oils in aromatic plants [11] and of secondary metabolites in roots [12–14]. Moreover, in alfalfa (*Medicago sativa* L.), barrel medic (*Medicago truncatula*), red clover (*Trifolium pratense*) and soybean (*Glycine max* L.) AM colonization increases flavonoid concentration [15–20]. In strawberry fruits, *Glomus intraradices* colonization increase cyanidin-3-glucoside concentration [21]. In addition, inoculation with AM fungi was shown to improve the quality of some crops such as artichoke [22] tomato [23,24] and maize [25], increasing the concentration of antioxidant molecules. Improved food quality can positively impact human health and reduce the costs for health care [26]. Increased quality, in terms of taste and nutritional value, can also become an additional target in agriculture, since, in recent years, consumers have sharpened their attention on all the aspects regarding the quality of foods and agricultural products in relation to health and environmental concerns.

Plant Growth-Promoting Bacteria (PGPB) improve plant nutrition, and support plant development under natural or stressed environments. This beneficial activity on plant growth can be exploited in order to realize cultivation protocols based on reduced amount of fertilizers and pesticides [27–29].

The mechanisms responsible for the stimulation of plant growth activated by PGPB can be both direct and indirect and involve: the biosynthesis of plant signaling molecules; the reduction of stress-ethylene in plants; the improvement of nutrient uptake (via fixation of N_2_ and phosphate solubilization); the synthesis of siderophores, antibiotics, enzymes and fungicidal molecules and niche competition, resulting in antagonistic activity against phytopathogenic microorganisms [30].

Since both AM fungi and PGPB can improve plant nutrition, their use in agriculture might result in reduced chemical inputs. High levels of fertilization, frequently occurring in intensive agriculture, negatively impact mycorrhizal colonization [10], constitute a significant cost for the producers and are a potential cause of eutrophication and pollution [31].

Therefore, the aim of this work was to demonstrate that the use of a consortium of AM fungi in combination with selected and tested *Pseudomonas* strains, in conditions of reduced fertilization (in particular phosphorus and nitrogen supply) induced an increased concentration of anthocyanins in strawberry fruits.

## 2. Results and Discussion

### 2.1. Anthocyanins Concentration in Strawberry Fruits

**Figure 1:**
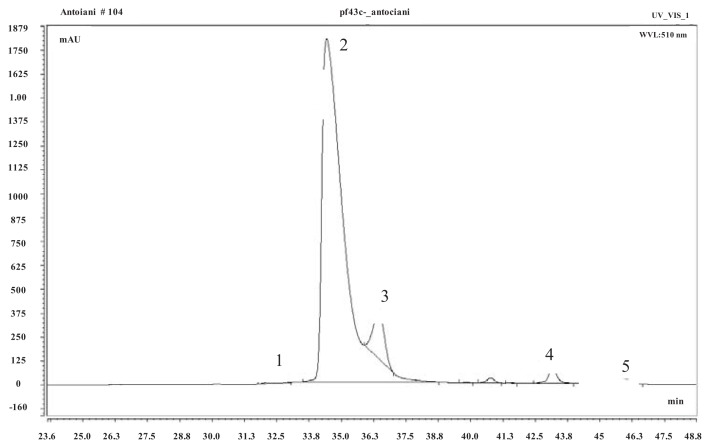
High performance liquid chromatography (HPLC) chromatogram showing the peaks of the anthocyanins detected in strawberry fruits.

The high performance liquid chromatography (HPLC) chromatogram displayed six peaks. Of these, five were identified as anthocyanins (Figure 1) on the basis of retention time and literature data; their identities were then confirmed by MS analysis.

The five anthocyanins identified in the strawberry fruits were cyanidin 3-glucoside (peak 1), pelargonidin 3-glucoside (peak 2), pelargonidin 3-rutinoside (peak 3), pelargonidin malonyl glucoside (peak 4), and pelargonidin acetyl glucoside (peak 5). In general, pelargonidin 3-glucoside was by far the most abundant identified anthocyanin, followed by pelargonidin 3-rutenoside.

**Figure 2:**
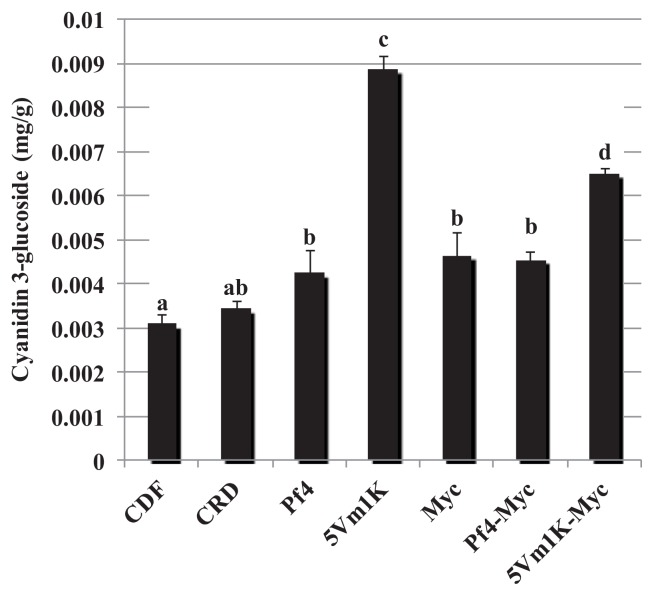
Mean (±standard error) cyanindin 3-glucoside concentration (mg/g) measured in strawberry fruits. Different letters indicate statistically significant differences between treatments. Plant treatments are identified as follows: **CDF**: Control 100, uninoculated plants with traditional fertilization; **CRD**: Control 70, uninoculated plants with 70% of the traditional fertilization; **Pf4**: plants inoculated with *P. fluorescens* Pf4 with 70% of the traditional fertilization; **5Vm1K**: plants inoculated with *Pseudomonas* sp. 5Vm1K with 70% of the traditional fertilization; **Myc**: plants inoculated with AM fungi and with 70% of the traditional fertilization; **Pf4-Myc**: plants inoculated with AM fungi and with *P. fluorescens* Pf4 with 70% of the traditional fertilization; **5Vm1K-Myc**: plants inoculated with AM fungi and with *Pseudomonas* sp. 5Vm1K with 70% of the traditional fertilization.

Any inoculation increased the concentration of cyanidin 3-glucoside compared to CFD (Figure 2), but strain *Pseudomonas* sp. 5Vm1K was the most effective of all microorganisms: when inoculated alone cyanidin concentration was three times that of controls, while in combination with AM fungi it promoted the doubling of this anthocyanin.

**Figure 3:**
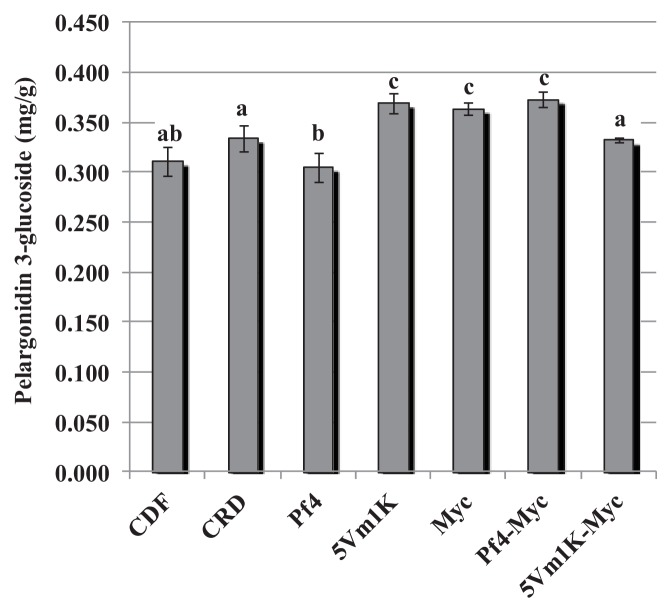
Mean (±standard error) pelargonidin 3-glucoside concentration (mg/g) measured in strawberry fruits. Different letters indicate statistically significant differences between treatments. Plant treatments are identified as follows: **CDF**: Control 100, uninoculated plants with traditional fertilization; **CRD**: Control 70, uninoculated plants with 70% of the traditional fertilization; **Pf4**: plants inoculated with *P. fluorescens* Pf4 with 70% of the traditional fertilization; **5Vm1K**: plants inoculated with *Pseudomonas* sp. 5Vm1K with 70% of the traditional fertilization; **Myc**: plants inoculated with AM fungi and with 70% of the traditional fertilization; **Pf4-Myc**: plants inoculated with AM fungi and with *P. fluorescens* Pf4 with 70% of the traditional fertilization; **5Vm1K-Myc**: plants inoculated with AM fungi and with *Pseudomonas* sp. 5Vm1K with 70% of the traditional fertilization.

**Figure 4:**
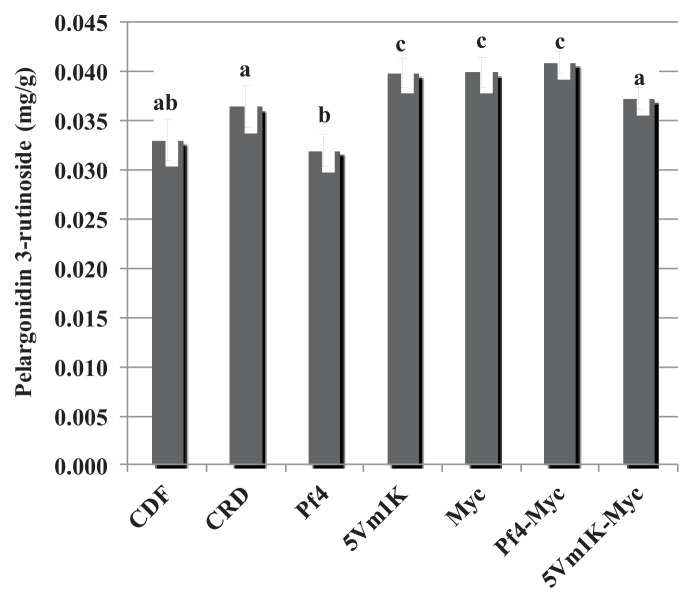
Mean (±standard error) pelargonidin 3-rutinoside concentration (mg/g) measured in strawberry fruits. Different letters indicate statistically significant differences between treatments. Plant treatments are identified as follows: **CDF**: Control 100, uninoculated plants with traditional fertilization; **CRD**: Control 70, uninoculated plants with 70% of the traditional fertilization; **Pf4**: plants inoculated with *P. fluorescens* Pf4 with 70% of the traditional fertilization; **5Vm1K**: plants inoculated with *Pseudomonas* sp. 5Vm1K with 70% of the traditional fertilization; **Myc**: plants inoculated with AM fungi and with 70% of the traditional fertilization; **Pf4-Myc**: plants inoculated with AM fungi and with *P. fluorescens* Pf4 with 70% of the traditional fertilization; **5Vm1K-Myc**: plants inoculated with AM fungi and with *Pseudomonas* sp. 5Vm1K with 70% of the traditional fertilization.

**Figure 5:**
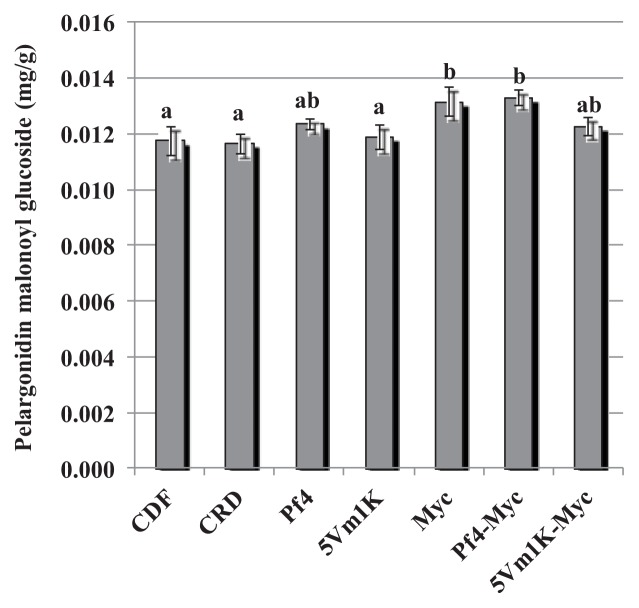
Mean (±standard error) pelargonidin malonyl glucoside concentration (mg/g) measured in strawberry fruits. Different letters indicate statistically significant differences between treatments. Plant treatments are identified as follows: **CDF**: Control 100, uninoculated plants with traditional fertilization; **CRD**: Control 70, uninoculated plants with 70% of the traditional fertilization; **Pf4**: plants inoculated with *P. fluorescens* Pf4 with 70% of the traditional fertilization; **5Vm1K**: plants inoculated with *Pseudomonas* sp. 5Vm1K with 70% of the traditional fertilization; **Myc**: plants inoculated with AM fungi and with 70% of the traditional fertilization; **Pf4-Myc**: plants inoculated with AM fungi and with *P. fluorescens* Pf4 with 70% of the traditional fertilization; **5Vm1K-Myc**: plants inoculated with AM fungi and with *Pseudomonas* sp. 5Vm1K with 70% of the traditional fertilization.

**Figure 6:**
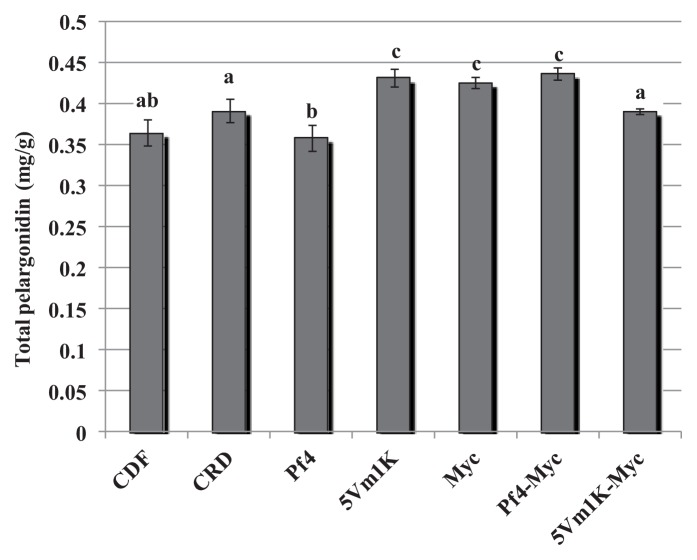
Mean (±standard error) total pelargonidin concentration (mg/g) measured in strawberry fruits. Different letters indicate statistically significant differences between treatments. Plant treatments are identified as follows: **CDF**: Control 100, uninoculated plants with traditional fertilization; **CRD**: Control 70, uninoculated plants with 70% of the traditional fertilization; **Pf4**: plants inoculated with *P. fluorescens* Pf4 with 70% of the traditional fertilization; **5Vm1K**: plants inoculated with *Pseudomonas* sp. 5Vm1K with 70% of the traditional fertilization; **Myc**: plants inoculated with AM fungi and with 70% of the traditional fertilization; **Pf4-Myc**: plants inoculated with AM fungi and with *P. fluorescens* Pf4 with 70% of the traditional fertilization; **5Vm1K-Myc**: plants inoculated with AM fungi and with *Pseudomonas* sp. 5Vm1K with 70% of the traditional fertilization.

The effect of bacteria and AM fungi was similar for pelargonidin 3-glucoside and pelargonidin 3-rutinoside: 5Vm1k, AM fungi and Pf4 + AM fungi significantly increased the concentration of the two anthocyanins compared to CFD and CRD (Figures 3 and 4). In the case of pelargonidin malonyl glucoside, only AM fungi and the combined inoculation of Pf4 and AM fungi were able to induce a significant increase of the molecule concentration (Figure 5). Values for pelargonidin acetyl glucoside did not show any significant variation (not shown). Total pelargonidin concentration is shown in Figure 6.

### 2.2. Mycorrhizal Colonization

At the end of the experiment the extent of AM colonization (M%) ranged between 2% and 4%. Traces of mycorrhizal colonization (M% always < 0.15) were detected in uninoculated plants. Roots of plants inoculated only with the AM fungi were colonized at a low extent (M% 3.0 ± 0.6). Co-inoculation AM fungi and the two bacterial strains resulted in similar values: 2.0 + 0.4 for *P. fluorescens* Pf4 and 4.1 ± 0.7 for *Pseudomonas* sp. 5Vm1K.

### 2.3. Nitrogen and Phosphorus Concentrations in the Plant Organs

Low nutrient supply resulted in a significant reduction of nitrogen concentration in leaves but not in the other organs of uninoculated plants (Table 1).

In roots, reduction of the nitrogen concentration was observed only in plants inoculated with AM fungi, alone or in combination with the strain 5Vm1K. Strain Pf4 and AM fungi increased leaf nitrogen concentration compared to that measured in CRD plants, while the strain 5Vm1K induced lower nitrogen concentration in leaves compared to CDF plants. Co-inoculation of each pseudomonad with AM fungi lead to a reduced amount of nitrogen in leaves compared to that recorded in plants cultivated following the standard fertilization protocol.

The reduced fertilization resulted in a decrease of phosphorus concentration in all plant organs of uninoculated plants (Table 2). Inoculation with any microorganism combination generally increased phosphorus concentration in the plant organs, often restoring (and sometimes exceeding) the values observed in CDF plants; the only exceptions were the leaves of Pf4-Myc plants and the roots of Pf4 plants.

### 2.4. Discussion

AM fungi are known to affect the plant secondary metabolism in roots and in shoots. For instance, in roots the flavonoid concentration is increased by AM symbiosis [15–18,20], while the concentration of essential oils is enhanced in several species [32–34].

Also PGPB can impact plant secondary metabolism. For example, it has been shown that *Azospirillum* sp. is able to modulate the phenolic compounds in rice [35], and that *Exiguobacterium oxidotolerans* increases the concentration of bacoside-A in *Bacopa monnieri* [36]. In *Arabidopsis*, *Pseudomonas fluorescens* SS101 is able to trigger defense responses promoting the biosynthesis of camalexing and glucosinolates [37].

In *Artemisia annua* it has been found that double inoculation with the AM fungus *Glomus mosseae* and *Bacillus subtilis* strain Daz26 can modulate artemisin content with synergistic effects [38].

The effect of AM colonization on the concentration of anthocyanins was previously measured in strawberry fruits by Castellanos-Morales *et al.* [21], who showed, for the first time, that symbiosis induces an increase in cyanidin 3-glucoside (and in that of some other phenolics). However, our results differ from those of this group in some aspects. Firstly, we inoculated plants with AM fungi and/or with two PGPB strains. Secondly, we have also measured the concentration of three additional forms of pelargonidin; although it has been found that pelargonidin 3-glucoside and cyanidin 3-glucoside represent the two main forms of anthocyanins in strawberry fruits (while other peaks in HPLC were described as unknown [39]), we could detect inoculation-dependent increases also for pelargonidin malonyl glucoside and pelargonidin 3-rutinoside. Finally, the concentrations detected by Castellanos-Morales [21] are much higher than those found in the present study, since they are about 10 times higher for pelargonidin 3-glucoside and 100 times higher for cyanidin 3-glucoside; these differences might depend on the different plant cultivars, since different concentration of anthocyanins can be associated both with genotype [40] and with environmental factors such as biotic interaction, light, irrigation, fertilization and cultivation methods therefore affecting anthocyanin concentration and antioxidant activity in strawberry fruits [41]; in addition concentrations might also depend on the different extent of mycorrhizal colonization observed in the present study and that of Castellanos-Morales and co-workers [21].

However, Crespo *et al.* [40] reported that pelargonidin-3-glucoside concentration ranges between 129 and 182 μg/g; while Aaby *et al.* [42] analysed 27 different strawberry cultivars and found pelargonidin 3-glucoside concentration values ranging between 69 and 389 μg/g; analysing five cultivars, Da Silva *et al.* [3] observed values between 162 and 468 μg/g; finally, Tulipani *et al.* [4] detected concentrations between 95.8 and 282.34 μg/g. In the present study, pelargonidin-3-glucoside concentration varied between 311 (in control plants) and 373 μg/g (in Pf4-M plants), values that fall in the range of the above-mentioned studies.

Co-inoculation with AM fungi and PGPB can result in synergistic effects on plants [43,44]. In the present study, positive interactions between AM fungi and PGPB and affecting anthocyanin concentration were not observed, while a decrease of anthocyanin concentration was found for the co-inoculation with AM fungi and 5Vm1K. Therefore, it is likely that this kind of effect can depend on the species and strains involved.

Strawberries are a particularly good source of antioxidants; it was found that strawberries have an antioxidant capacity 10-fold greater than that of many other fruits, including oranges, kiwi, grapefruit, grapes and mangos, among others [45]. Anthocyanins are in part responsible for the antioxidant power of strawberry fruits, together with ascorbic acid and a wide variety of phenolics, including hydroxybenzoic and hydroxycinnamic acid derivates, flavonols, flavanols, proanthocyanidins and hydrolysable tannins [46].

A certain correlation between diets rich in fruits and vegetables and a lower incidence of some major human chronic diseases, including several forms of cancer and cardiovascular disease, among others, has been observed in a wide variety of epidemiological studies [47]. These beneficial effects have been attributed to a wide variety of naturally occurring compounds that have not been traditionally regarded as nutrients, phenolics being one of the most important groups. Increased concentration of antioxidant molecules in some crops was previously found in artichoke [22] and tomato [23,24], resulting in products of higher nutraceutical value. This could impact on human health, on the cost of welfare and even on the revenue for the producer. Furthermore, if increased crop value can be reached reducing the chemical inputs (either fertilizers, as in the present study, or pesticides, due to the increased resistance of mycorrhizal plants towards pathogens [48–50]), environmental benefits can be envisaged as well.

Anthocyanins are also responsible for the red and blue colours of many fruits and berries such as chokeberry, black currant and strawberry. The quantitative analysis of anthocyanins in strawberries is important not only to assess their degree of maturity, but also due to the fact that they are responsible for their colour, and this attribute does influence a great deal of the consumer’s preferences [51].

Higher anthocyanin concentration following AM colonization might depend on the activation of a defence response in the plant. Indeed, pathogens and insects increase the concentration of anthocyanins and antioxidants in strawberry fruits as defence mechanisms in fruits [52]. However, nutritional factors might play an even more important role on the control of anthocyanin concentrations. Nitrogen concentrations in the nutrient solutions provided to strawberry modulates the amount of phenolic compounds in the fruits: at low nitrogen concentration total phenols decrease, as well as gallic acid and cyanidin 3-glucoside, while ellagic acid, kaempferol and quercetin increase, compared to those observed at high nitrogen concentration [21]. In tomato it was found that, in order to obtain fruits with a good colour, N supply should be kept as low as possible without reducing fruit yield, suggesting the existence of a nitrogen-dependent mechanism that controls the concentration of phenols in the fruit [53]. Actually, secondary plant metabolites lacking N in their structure, such as lycopene, phenolics and flavonols, are favoured under N-limiting conditions as long as photosynthetic activity is not simultaneously reduced, whereas nitrogen-containing compounds are favoured when N is readily available and not limiting to growth [54].

Phosphorus, in plants, has multifunctional roles as a constituent of nucleic acids or biomembranes. Furthermore, it is highly involved in the energy metabolism of cells and is therefore required for the biosynthesis of primary and secondary metabolites [55]. In tomato, phosphorus may increase the level of some phytochemicals like ascorbic acid, anthocyanins, flavonoids and lycopene [54]. In strawberry, AM fungi and rhizosphere microorganisms can modulate phosphorus concentration [56]).

The low levels of mycorrhizal colonization observed in this study might depend on two factors. On the one hand, the plants were grown at N and P concentrations that, even when fertilization was reduced, were not limiting for the plant growth, nor for the fruit yield [57]. It is well known that high concentration of P and N can limit mycorrhizal colonization [58]. In the present study, nitrogen concentration was always in the range (19–40 mg/g) that does not result in plant stress because of lack or excess of this nutrient [59,60]. On the other hand, “Selva” is a variety with a continuous flowering (and fruiting) habit. It is known that the extent of AM root colonization can be modulated according to phenology. Fruit production is a major sink for carbon, possibly decreasing the availability of carbohydrates for the fungus and therefore resulting in decreased colonization, in agreement with the observation that plants with reduced assimilate supply of roots (as a consequence of reduced phloem loading) decrease AM colonization [61].

The levels of mycorrhizal colonization observed in this study were low and this might depend on two factors. On the one hand, the plants were grown at N and P concentrations which, even when fertilization was reduced, were not limiting for the growth, nor for the fruit yield [57], of uninoculated plants. It is well known that high concentrations of P and N can limit mycorrhizal colonization [58]. In the present study, nitrogen concentration was always in the range (19–40 mg/g) that does not result in plant stress due to lack or excess of this nutrient [59,60]. On the other hand, “Selva” is a variety with a continuous flowering (and fruiting) habit. It is known that the extent of AM root colonization can be modulated according to phenology. Fruit production is a major sink for carbon, possibly decreasing the availability of carbohydrates for the fungus and therefore resulting in decreased colonization, in agreement with the observation that plants with reduced assimilate supply of roots (as a consequence of reduced phloem loading) decrease AM colonization [61]. In this respect, it can be added that during a field test with the same variety of strawberry, we monitored mycorrhizal colonization before and after the onset of flowering; after blooming, M% was about a half of the value observed before flowering [62].

## 3. Experimental Section

### 3.1. Plants and Microorganisms

Frigo plantlets of a commercial variety of strawberry (*Fragaria* × *ananassa* var. Selva) with continuous flowering habit were used.

A mycorrhizal inoculum consisting of sporocarps, spores, hyphae, and root fragments colonized by a number of AM fungi (all of them belonging to the genus *Glomus*), collected from an agricultural soil where potatoes and carrots were grown, was produced and provided by Mybasol s.r.l. (Alessandria, Italy).

Two bacterial strains were used to inoculate the plants, alone or in combination with the mycorrhizal inoculum. *Pseudomonas fluorescens* strain Pf4 (briefly: Pf4) was isolated from a woody soil located in Sassello (Savona, Italy). Pf4 synthesizes siderophores, is able to solubilize phosphates at neutral, acid and alkaline pH and produces the phytohormone IAA as reported in Berta *et al.* [25].

*Pseudomonas* sp. 5Vm1K (abbreviated: 5Vm1K) was isolated from the rhizosphere of *Vaccinium myrtillus* and grown in a larch woodland located in Bellino (CN, Italy) and characterized as described by Bona *et al.* [57].

### 3.2. Experimental Design and Plant Growth

Greenhouse assays were conducted to evaluate the effect of the microorganisms on plant growth, flower and fruit production, and quality.

Each strawberry frigo plantlet was transplanted into a 3 l plastic pot filled with mineral substrate “Terra Mediterranea” (Harpo S.p.A., Trieste, Italy) containing low concentrations of phosphorus and nitrogen.

For plant inoculation, bacteria were grown on tryptic soy agar (TSA, BD Difco, NJ, USA) at 28 °C for 48 h. Bacterial cells were scraped from the medium and suspended in MgSO_4_ 0.1 M (Fluka, St. Louis, MO, USA), washed twice and finally resuspended in MgSO_4_·7H_2_O 0.1 M. Bacterial density of the suspension was assessed using a calibration curve assessed by turbidity (λ = 600 nm) and adjusted to 10^9^ CFU·mL^−1^. 5 mL of the bacterial suspensions were used to inoculate each treated plant at transplanting, and the inoculation was repeated after 20 days. The same amount of buffer was provided to uninoculated plants.

Mycorrhizal plants were inoculated with 100 mL of the above-described AM inoculums mixed with the substrate at transplanting.

All plants were fertilized weekly with a solution that contained about 70% of N and P_2_O_5_ used in conventional practise (N 3.145 g/pt; P_2_O_5_ 1.169 g/pt; K_2_O 4.081 g/pt; CaO 1.763 g/pt; MgO 0.414 g/pt), except one set of plants that was grown at the full dose of nutrients (N 4.605 g/pt; P_2_O_5_ 1.670 g/pt; K_2_O 4.940 g/pt; CaO 3.785 g/pt; MgO 0.815 g/pt); all plants received the following micronutrient: B 0.113 g/pt; Cu 0.225 g/pt; Fe 2.925 g/pt; Mn 0.900 g/pt; Mo 0.011 g/pt; Zn 0.675 g/pt. Fertilizers were provided by Greenhas Italia (Canale, CN, Italy). On alternate days plants were fed with tap water. Therefore, the experimental design included seven treatments, each one consisting of 10 plants: CFD: uninoculated (control) plants fertilized according to the conventional practise; CRD: uninoculated (control) plants with reduced fertilization; Pf4: plants inoculated with *P. fluorescens* strain Pf4 and grown with reduced fertilization; 5Vm1K: plants inoculated with *Pseudomonas* sp. strain 5Vm1K and grown with reduced fertilization; Myc: plants inoculated with the AM consortium and grown with reduced fertilization; Pf4-Myc: plants inoculated with *P. fluorescens* strain Pf4 and the AM consortium, and grown with reduced fertilization; 5Vm1K-Myc: plants inoculated with *Pseudomonas* strain 5Vm1K and the AM consortium, and grown with reduced fertilization.

The experiment was performed between April and September 2011, in greenhouse and lasted 22 weeks. Temperature and humidity were monitored continuously by means of a thermo-hygrograph.

### 3.3. Fruit Collection and Plant Harvest

Fruits were harvested from April to September when most of their surface reached a full red colour, from April to September. Sepals were discarded and only fruit flesh was used for further analyses [63]. Harvested fruits were immediately frozen in liquid nitrogen and stored at −80 °C for biochemical analyses.

After 22 weeks of growth, the plants were harvested. The roots were separated from the aerial part and washed with deionised water. Roots from each plant were stored in 70% ethanol for mycorrhizal assay.

### 3.4. Mycorrhizal Colonization

Forty randomly chosen 1 cm-long pieces were cut from each root system, fixed in 70% ethanol, and then stored at 4 °C until analysis. Root pieces were cleared in 10% KOH for 45 min at 60 °C, stained with 1% methyl blue in lactic acid and mounted on a slide. Mycorrhizal colonization was estimated according to Trouvelot *et al.* [64].

### 3.5. Fruit Analyses

The total fruit production of each treatment was divided into three parts according to the harvest time (roughly corresponding to the months of June, July and August). The concentration of two anthocyanins (pelargonidin and cyanidin) was measured on fruit homogenates.

Anthocyanins were extracted and separated according to Comandini *et al.* [65] with some modifications. 10 grams of fruit homogenate were mixed with 10 mL of methanol (Sigma-Aldrich, St. Louis, MO, USA) for 1 min. The extract was centrifuged at 13,000 rpm for 30 min at 10 °C. The supernatant was collected and a second extraction was performed on the sample residue with 10 mL of 95% aqueous methanol. The solid residue and the hydro-alcoholic were first homogenized and then centrifuged at 13,000 rpm for 10 min at 10 °C. The supernatants of the two extractions were combined and concentrated in a speedvac (concentrator 5301, Eppendorf, Hamburg, Germany). The concentrated extract was dissolved in 5 mL acidified water (3% formic acid) and run on a SPE Discovery C18 Cartridge (500 mg/6 mL) (Supelco, Bellefonte, PA, USA), previously activated with methanol followed by HPLC-grade water and by 3% aqueous formic acid. Anthocyanin and other phenolic compounds were recovered with 2 mL of methanol containing 3% formic acid. Methanol extracts were filtered through 0.22 μm filters and injected in a HPLC instrument (Dionex, Sunnyvale, CA, USA) consisting of a solvent delivery system (Ultimate 3000 pump LPG-3400A) with an integrated vacuum degasser and mixing chamber, a detector (UVD-3000), an auto-sampler (WPS-3000TSL Analytical), a pump control and a data analysis system (Chromeleon 6.70 SP7). Anthocyanins were separated with a pre-column (or guard column- Aphera C18 polymer 15 cm × 4.6 mm, 5 μm) and an analytical column (Aphera C18 polymer 25 cm × 4.6 mm, 5 μm) at a flow rate of 0.5 mL/min, at 30 °C. The separation of anthocyanin was performed with a mobile phase composed of a solvent A (2.5% *v*/*v* formic acid in HPLC-grade water) and a solvent B (2.5% *v*/*v* formic acid in HPLC-grade methanol). The following linear elution gradient was employed: from the beginning to 5 min, 85% solvent A and 15% Solvent B; from 5 to 20 min, solvent A increased up to 65% and held constant until 25 min; from 25 to 35 min solvent A decreased to 50% and then was kept constant until 45 min; from 45 to 50 min solvent A decreased to 34%; finally, the columns were washed and restored to 85% solvent A. The total run time was 80 min. Anthocyanins were detected at 510 nm (injection volume 20 μL).

Each anthocyanin was quantified according to Chandra *et al.* [66]. Commercially available standards of pelargonidin chloride and cyaniding chloride were used as stock solutions for generating a five-point calibration curve.

The amounts of each anthocyanin measured in the samples were expressed as A × DF × CF/SWT where A = amount of anthocyanin expressed as external standard equivalent from the calibration curve (mg/mL); DF = dilution factor; CF = molecular white correction factor to convert individual anthocyanin calculated as the external standard equivalents to their respective forms; SWT = initial sample white. The concentration of anthocyanins was referred to the fruit fresh weight.

### 3.6. MS Anthocyanin Identification

The anthocyanin extract was also used for nano-LC/ESI-MS (liquid chromatography/electron spray ionization-mass spectrometry) analysis. Fractions (corresponding to peaks 1, 2, 3, 4 and 5 in Figure 1) were concentrated in a speedvac Concentrator 5301.

Each fraction was resuspended in 5 μL 0.1% formic acid before nanoHPLC-electrospray mass spectrometry (nanoLC-ESIMS/MS) analysis. After injection the sample was pre-concentrated and washed onto a trapping cartridge (C18, 0.3 cm × 5 mm, DIONEX, LC Packings) using a Famos autosampler (DIONEX, LC Packings, Amsterdam, the Netherlands) at 40 μL/min of 0.1% formic acid.

Using an Ultimate solvent delivery system (DIONEX, LC Packings, Amsterdam, the Netherlands), a linear gradient of acetonitrile (0.1% formic acid) from 20% to 95% over 85 min at flow rate of ~300 nL/min was used.

Quadrupole time of flight mass spectrometry (Q-TOF-MS) and quadrupole time of flight mass spectrometry/mass spectrometry (Q-TOF-MS/MS) analyses were performed using a hybrid quadruple-time of flight QStar XL (AB SCIEX, Framingham, MA, USA) equipped with a nanospray source operating in the positive ion mode. The needle voltage was 1800V. Data were processed using Analyst-QS V2.0 software provided with the spectrometer.

Key parameter settings were as follows: ionspray voltage (IS) = 1900 V, curtain gas (CUR) = 25, declustering potential (DP) = 80 V, focusing potential (FP) = 280 V, collision gas setting (CAD) = 3 for nitrogen gas, DP2 = 25. Data were acquired using information-dependent acquisition (IDA) mode with Analyst QS software (AB-SCIEX). Mass range 100–1500 m/z for MS and 10–1800 *m*/*z* for MS/MS.

### 3.7. Phosphorus and Nitrogen Concentration in the Plant Organs

Phosphorus and nitrogen concentrations in strawberry shoot and roots were determined. Aliquots (0.5 g dry weight) of each sample were digested in 6 mL of 65% nitric acid (Sigma-Aldrich) using a MARS 5 microwave oven (CEM, Matthews, CA, USA). Digested samples were analysed by inductively coupled plasma-optical emission (IRIS Advantage ICAP series DUO HR, Thermo Jarrell Ash, Franklin, MA, USA) and inductively coupled plasma-MS (Plasma QUAD 3, VG Elemental Europe, Cedex, France). Certified standards of analysed metal and acid blanks were run with all sample series for quality control. The phosphorus and nitrogen concentrations are given as percentage of dry weight.

### 3.8. Statistical Analysis

Statistical analyses were performed with StatView 4.5 (Abacus Concepts, Barkeley, CA, USA). Data were analyzed by two-way ANOVA using “fungus” (consisting of two levels: no fungal inoculation, and inoculation with the AM fungi) and “bacterium” (three levels: no bacterial inoculation, inoculation with Pf4, and inoculation with 5Vm1K) as factors. A one-way ANOVA, using “treatment” as factor, followed by Fisher’s probable least-squares difference test with cut-off significance at *p* < 0.05 was used to assess differences between treatments.

## 4. Conclusions

Inoculation of strawberry with AM fungi and PGPB resulted in increased concentration of the two main anthocyanins in the fruit produced by plants cultivated under conditions of reduced fertilization. In addition, three forms of pelargonidin were identified. Overall, our results suggest that the common fertilization practise provides nutrients in excess. Lower concentration of nutrients and inoculation with soil microorganisms can result in healthier fruits, with a higher concentration of antioxidant molecules, consistent with the improved nutritional quality of vegetables, as previously reported [23,24,67]). At the same time, data concerning the same plants used for this study showed that yield was not affected, even at reduced fertilization [56].

## Figures and Tables

**Table 1:** Mean nitrogen concentration (±standard error) in plant organs, given as percentage of dry weight. Different letters indicate significantly different means.

Treatments 1	Leaves	Roots
CDF	2.75 ± 0.08 a	1.46 ± 0.09 a
CRD	2.51 ± 0.09 b	1.48 ± 0.11 a
Pf4	2.79 ± 0.06 a	1.29 ± 0.09 a
5Vm1K	2.44 ± 0.38 b	1.44 ± 0.12 a
Myc	2.78 ± 0.11 a	1.18 ± 0.14 b
Pf4-Myc	2.58 ± 0.05 b	1.50 ± 0.13 a
5Vm1K-Myc	2.58 ± 0.10 b	1.11 ± 0.11 b
*p*-value	0.0077	0.0009

1 Plant treatments; CDF: Control 100, uninoculated plants with traditional fertilization; CRD: Control 70, uninoculated plants with 70% of the traditional fertilization; Pf4: plants inoculated with *P. fluorescens* Pf4 with 70% of the traditional fertilization; 5Vm1K: plants inoculated with *Pseudomonas* sp. 5Vm1K with 70% of the traditional fertilization; Myc: plants inoculated with AM fungi and with 70% of the traditional fertilization; Pf4-Myc: plants inoculated with AM fungi and with *P. fluorescens* Pf4 with 70% of the traditional fertilization; 5Vm1K-Myc: plants inoculated with AM fungi and with *Pseudomonas* sp. 5Vm1K with 70% of the traditional fertilization.

**Table 2:** Mean phosphorous concentration (±standard error) in plant organs, given as percentage of dry weight. Different letters indicate significantly different means.

Treatments 1	Leaves	Roots
CDF	0.51 ± 0.04 a	0.41 ± 0.04 a
CRD	0.38 ± 0.03 b	0.33 ± 0.02 b
Pf4	0.48 ± 0.03 a	0.34 ± 0.02 ab
5Vm1K	0.47 ± 0.02 a	0.50 ± 0.07 a
Myc	0.42 ± 0.03 c	0.39 ± 0.04 a
Pf4-Myc	0.39 ± 0.03 b	0.40 ± 0.03 a
5Vm1K-Myc	0.47 ± 0.03 a	0.45 ± 0.07 a
*p*-value	<0.0001	0.0039

1 Plant treatments; CDF: Control 100, uninoculated plants with traditional fertilization; CRD: Control 70, uninoculated plants with 70% of the traditional fertilization; Pf4: plants inoculated with *P. fluorescens* Pf4 with 70% of the traditional fertilization; 5Vm1K: plants inoculated with *Pseudomonas* sp. 5Vm1K with 70% of the traditional fertilization; Myc: plants inoculated with AM fungi and with 70% of the traditional fertilization; Pf4-Myc: plants inoculated with AM fungi and with *P. fluorescens* Pf4 with 70% of the traditional fertilization; 5Vm1K-Myc: plants inoculated with AM fungi and with *Pseudomonas* sp. 5Vm1K with 70% of the traditional fertilization.

## Abbreviations

CDF, Control 100: uninoculated plants with traditional fertilization
CRD: Control 70, uninoculated plants with 70% of the traditional fertilization
Pf4: plants inoculated with *P. fluorescens* Pf4 with 70% of the traditional fertilization
5Vm1K: plants inoculated with *Pseudomonas* sp. 5Vm1K with 70% of the traditional fertilization
Myc: plants inoculated with AM fungi and with 70% of the traditional fertilization
Pf4-Myc: plants inoculated with AM fungi and with *P. fluorescens* Pf4 with 70% of the traditional fertilization
5Vm1K-Myc: plants inoculated with AM fungi and with *Pseudomonas* sp. 5Vm1K with 70% of the traditional fertilization
